# Post-injury treatment with 7,8-dihydroxyflavone attenuates white matter pathology in aged mice following focal traumatic brain injury

**DOI:** 10.1016/j.neurot.2024.e00472

**Published:** 2024-10-20

**Authors:** Georgios Michalettos, Fredrik Clausen, Elham Rostami, Niklas Marklund

**Affiliations:** a Department of Clinical Sciences Lund, Neurosurgery, Lund University, Lund, Sweden; b Department of Medical Sciences, Section of Neurosurgery, Uppsala University, Uppsala, Sweden; c Department of Neuroscience, Karolinska Institute, Stockholm, Sweden; d Department of Clinical Sciences Lund, Neurosurgery, Lund University and Lund University Hospital, Lund, Sweden

**Keywords:** Traumatic brain injury, High age, 7,8-DHF, White matter, Oligodendroglia, Axonal injury

## Abstract

Traumatic brain injury (TBI) is a major cause of morbidity and mortality, not least in the elderly. The incidence of aged TBI patients has increased dramatically during the last decades. High age is a highly negative prognostic factor in TBI, and pharmacological treatment options are lacking. We used the controlled cortical impact (CCI) TBI model in 23-month-old male and female mice and analyzed the effect of post-injury treatment with 7,8 dihydroxyflavone (7,8-DHF), a brain-derived neurotrophic factor (BDNF)-mimetic compound, on white matter pathology. Following CCI or sham injury, mice received subcutaneous 7,8-DHF injections (5 ​mg/kg) 30 ​min post-injury and were sacrificed on 2, 7 or 14 days post-injury (dpi) for histological and immunofluorescence analyses. Histological assessment with Luxol Fast Blue (LFB)/Cresyl Violet stain showed that administration of 7,8-DHF resulted in preserved white matter tissue at 2 and 7 dpi with no difference in cortical tissue loss at all investigated time points. Treatment with 7,8-DHF led to reduced axonal swellings at 2 and 7 dpi, as visualized by SMI-31 (Neurofilament Heavy Chain) immunofluorescence, and reduced number of TUNEL (Terminal deoxynucleotidyl transferase dUTP nick end labelling)/CC1-positive mature oligodendrocytes at 2 dpi in the perilesional white matter. Post-injury proliferation of Platelet-derived Growth Factor Receptor (PDGFRα)-positive oligodendodrocyte progenitor cells was not altered by 7,8-DHF. Our results suggest that 7,8-DHF can attenuate white matter pathology by mitigating axonal injury and oligodendrocyte death in the aged mouse brain following TBI. These data argue that further exploration of 7,8-DHF towards clinical use is warranted.

## Introduction

Traumatic brain injury (TBI) is associated with high mortality and persistent morbidity, not least in the young [[1], [2], [3]]. However, in recent decades, there has been a vast increase in the TBI incidence among the elderly population, mainly due to falls [[4], [5], [6], [7], [8]]. Since the life expectancy is increasing in most countries and an increasing proportion of elderly lead an active lifestyle, the number of TBIs in the geriatric cohorts is expected to increase further. High age is an established negative prognostic risk factor for a poor outcome following TBI [9]. Co-morbidities that include the presence of anti-thrombotic medications at time of injury contribute [10], but there are plausibly many age-specific differences in the biological response to a TBI. To date, prevention is a key in TBI management since current neurosurgical interventions and medical care are insufficient for providing a good outcome in a majority of elderly patients [11,12]. Importantly, there are no pharmacological compounds shown to improve TBI outcome regardless of the age of the patients. Enhanced translatability of preclinical findings is crucial in developing more effective treatment strategies, ensuring that novel therapeutics are applicable and of benefit to the elderly population. However, preclinical TBI studies in aged animals remain scarce [[13], [14], [15], [16], [17], [18], [19]].

Much of the cognitive and behavioral sequelae observed after TBI is related to white matter pathology, involving injury to the axons as well as to other white matter components such as oligodendroglia [[20], [21], [22], [23], [24], [25], [26], [27], [28], [29]]. Disrupted white matter integrity may lead to impaired functional connectivity of the neuronal networks, resulting in cognitive and motor deficits as well as long-lasting behavioral disturbances [[30], [31], [32], [33], [34]]. Pharmacological interventions which can preserve brain structure and promote plasticity may show promise for improving neurological outcome post-TBI. In an increasing number of preclinical studies, TBI-induced white matter changes have been evaluated although such studies have not been performed on aged animals [[35], [36], [37], [38], [39], [40]].

The use of neurotrophic factors has received much interest in TBI due to their potential for promoting neuronal survival, regeneration, and functional recovery [41]. The Brain-Derived Neurotrophic Factor (BDNF), acting primarily by binding to the Tropomyosin receptor kinase B (TrkB) and the p75 Neurotrophin Receptor (p75-NTR), has been found to preserve and restore lost neurological function [42,43]. However, factors such as pharmacokinetics, half-life, stability, and ability to penetrate the blood-brain barrier when administered exogenously, have posed significant challenges to the clinical utilization of BDNF [[43], [44], [45]]. Among the scientific exploration of small molecule compounds which can potentially mimic the desired properties of the brain's endogenous molecules, 7,8- dihydroxyflavone (7,8-DHF), a naturally occurring flavonoid, may act on the central nervous system (CNS) by increasing plasticity and mitigating the development of neurodegenerative diseases.

The flavonoid 7,8-DHF is a BDNF-mimetic small molecule which activates the TrkB receptor and efficiently crosses the blood-brain barrier [46,47]. In TBI, several studies found benefits of 7,8-DHF in promoting neurological recovery in experimental diffuse and focal rodent TBI models [43,[48], [49], [50], [51]]. These effects have been attributed to attenuated neuronal cell death and increased expression of plasticity-related proteins. However, the aging brain is characterized by low capacity for plasticity and regeneration [52,53], and the functionality of the BNDF/TrkB pathway may be impaired in the aging brain [[54], [55], [56], [57]]. The beneficial effects of DHF were convincingly shown in young mice in previous studies, but its efficacy may diminish in the aging rodent brain. Our aim was to determine whether post-injury treatment with 7,8-DHF could positively influence injury to white matter components in the aged, 23-month-old mouse brain using an experimental model of focal TBI.

## Materials and Methods

### Animals and treatment

Sixty male and female C57Bl/6 mice aged 23 months, were used. The experiments were designed using the PREPARE protocol (https://norecopa.no/prepare), were approved by the local animal ethics committee (Permit no: 5.8.18-00148/2020, addendum 5.8.18-19167/2022) and were performed according to the regulations of the Swedish Board of Agriculture. Animals were housed in a controlled environment of 12-h light-dark cycle with *ad libitum* access to food and water. Of these 60 animals, 27 were female (22-36 ​g) and 33 were male (25-43 ​g). Mice were initially selected to receive the controlled cortical impact injury (CCI) or sham injury according to the “Randomized Block” approach where each mouse cage represents an independent “block”. In total, 46 mice received the CCI (CCI-mice) from which 15 animals (7 males and 8 females) were allocated to the survival endpoint of 2 days post-injury (dpi), 16 animals (8 males and 8 females) to 7 dpi and 15 animals (7 males and 8 females) to 14 dpi. Fourteen mice underwent sham injury (sham- injured control mice) and were allocated to the survival endpoint of 2 dpi.

**Fig. 1:**
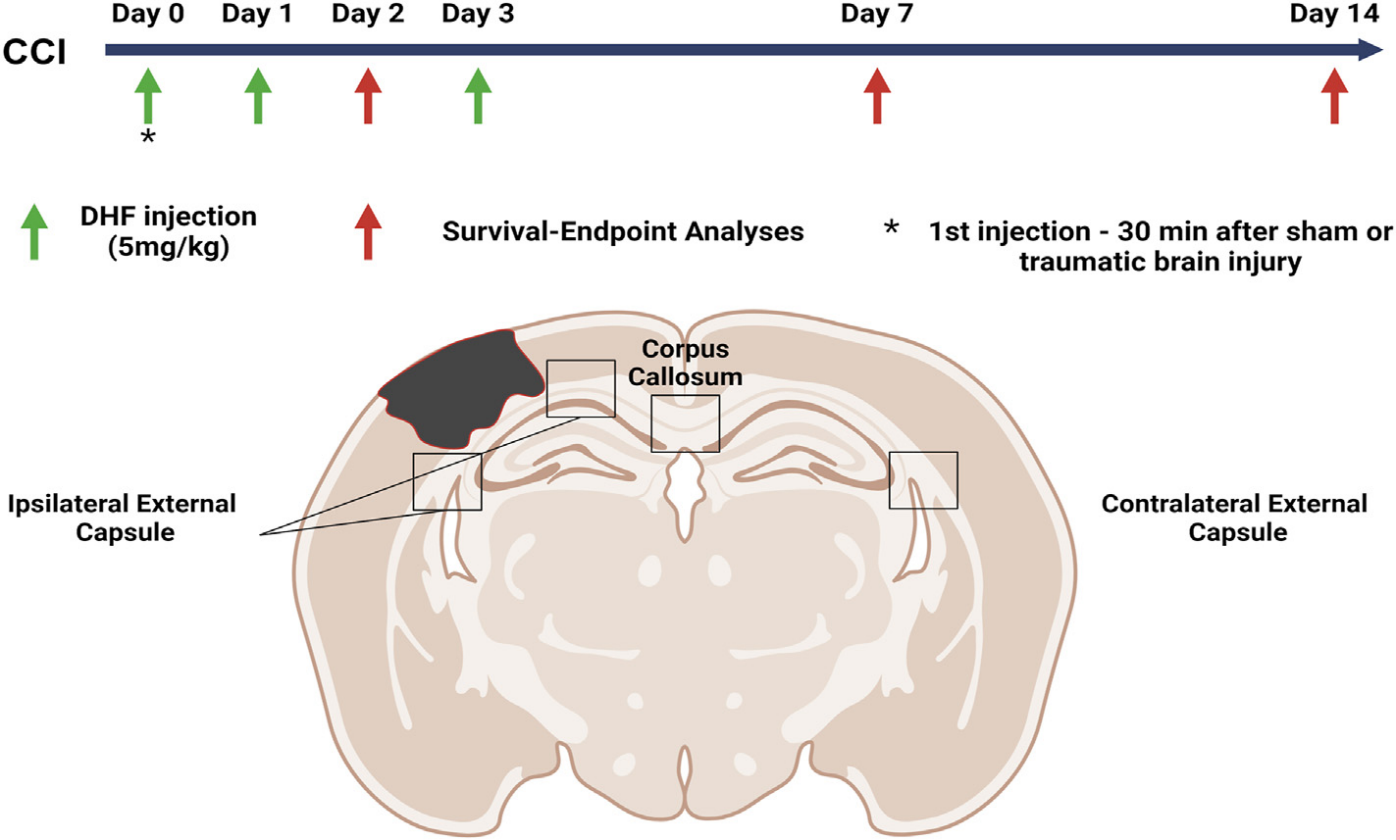
**Experimental Design**. Mice underwent focal traumatic brain injury (TBI) using the CCI model. All animals received subcutaneous 7,8-DHF injections or saline at 30 ​min after surgery and at 1 dpi and had survival time points of either at 2, 7 or 14 dpi. In addition, CCI mice which were allocated to survival for 7 or 14 dpi received a third 7,8-DHF injection at 3 dpi. Sham-injured mice survived until 2 dpi and received 2 DHF or saline injections. The graphical illustration of the mouse brain coronal section depicts the lesion along with the regions of interest (ROI) focused on this study. The graphical illustration was made using BioRender. Dpi- days post-injury, CCI- controlled cortical impact.

Mice were then randomly selected to receive either 7,8-DHF or saline following sham or CCI surgery. Following TBI or sham injury, 7,8-DHF or saline was administered subcutaneously (s.c) 30 ​min after injury. Injections were repeated at 1 dpi for all survival endpoints and at 3 dpi for the survival endpoints of 7 and 14 dpi. Treatment with 7,8-DHF, dissolved in saline and injected s.c., was at a concentration of 5 ​mg/kg. In total, 7 CCI-mice treated with 7,8-DHF (CCI-DHF) (4 females and 3 males) and 8 CCI-mice treated with saline (CCI-NaCl) (4 females and 4 males) were sacrificed at 2 dpi, 8 CCI-DHF mice (4 females and 4 males) and 8 CCI-NaCl mice (4 females and 4 males) at 7 dpi and 8 CCI-DHF mice (4 females and 4 males) and 7 CCI-NaCl mice (4 females and 3 males) at 14 dpi. Regarding controls, 7 sham-DHF mice (2 females and 5 males) and 7 sham-NaCl mice (2 females and 5 males) were sacrificed at 2 dpi. An overview of the experimental design is shown in Fig. 1.

### Controlled cortical impact injury model

The controlled cortical impact injury (CCI) model or sham injury were performed according to methods previously published [58,59]. In brief, mice were initially anesthetized with 3.5 % isoflurane and fixated on the stereotactic frame. Anesthesia was maintained using 1.8 % isoflurane through a nose cone in a mixture of nitrous oxide and oxygen (70/30 ​%). Ophthalmic ointment (Viscotears, Bausch & Lomb, Stockholm, Sweden) was applied in order to prevent dryness of the animal's eyes. A heating pad was used throughout the surgery to maintain the animal's body temperature at 37 ​°C monitored by a rectal probe. Following a midline incision of the scalp, a 4-mm diameter craniotomy was created over the left parietotemporal cortex. Injury induction was done using a CCI-device (Impact One TM Leica Biosystems, Mölndal, Sweden) with injury parameters set to decompression depth of 0.8 ​mm, impact velocity of 4.0 ​m/s and a dwelling time of 100 ​ms. The diameter of the piston was 2.5 ​mm. Following injury, the craniotomy was put back, the wound was sutured, and the animal was placed in a cage with a heating pad until awakening. Sham-injured animals underwent the exact same procedure including the incision, the craniotomy and the replacement of the craniotomy, with the exception of the piston not being released. There was no mortality of the surgical procedure or the CCI injury, and no animals were excluded.

### Tissue acquisition

Mice received a 0.5 ​ml intraperitoneal injection with a fatal overdose of sodium pentobarbital (Euthasol Vet. 400 ​mg/ml, Dechra Veterinary Products, Shrewsbury, UK) at 2, 7 or 14 dpi. Thereafter, mice were transcardially perfused with 0.9 ​% saline and fixated with 4 ​% paraformaldehyde (PFA) (Merck, Darmstadt, Germany). Brains were removed and post-fixed overnight at 4 ​°C in 4 ​% PFA. Brains were, then, switched to 25 ​% sucrose solution for 48–72hr and sectioned on a freezing microtome with the use of dry ice. Serial coronal sections (20 ​μm thick) were cut and kept in anti-freeze cryoprotect solution (30 ​% ethyleneglycol & 30 ​% glycerol) at −20 ​°C for further use.

### Nissl/Luxol Fast Blue histology and tissue loss estimation

In order to distinguish the white and gray matter compartment of the lesion, we combined the Luxol Fast Blue histological stain with the use of Nissl stain as counterstain. Combined Luxol Fast Blue and Cresyl Violet (LFB/CV) stains were conducted using standard histological protocols. In brief, sections were rinsed with PBS, mounted on positively charged slides and let to dry overnight. Slides were incubated in 95 ​% alcohol and subsequently submerged in 0.1 ​% LFB overnight at 58 ​°C. Differentiation was done with 0.05 ​% Lithium Carbonate until satisfactory result was achieved. Slides were sufficiently re-hydrated in distilled water and incubated in 0.1 ​% CV at 50 ​°C for 45 ​min. Differentiation was done with 95 ​% ethanol. Slides were, then, moved to absolute ethanol, followed by xylene and mounted using Pertex (Histolab AB, Gothenburg, Sweden).

**Fig. 2:**
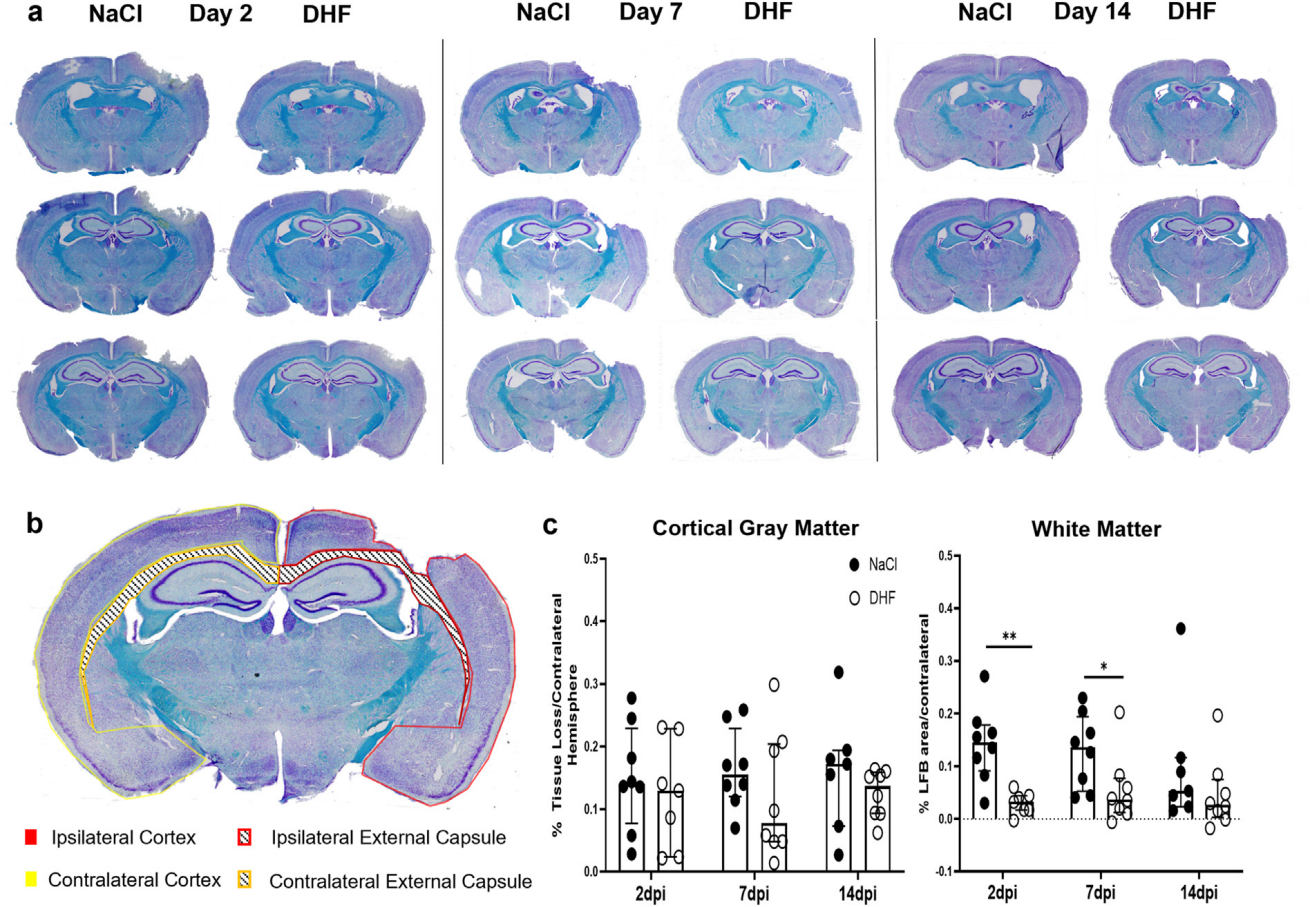
**7,8-DHF protects against white matter tissue loss without affecting cortical tissue loss**. **(A)** Representative Luxol Fast Blue/Cresyl Violet-stained sections from DHF-treated and saline-treated mice at 2, 7 and 14 dpi. **(B)** Coronal section containing the outlines of the gray and white matter areas analyzed for tissue loss assessment following CCI and treatment with 7,8-DHF. **(C)** Quantification of tissue loss percentage in the cortex and the white matter of DHF-treated and saline-treated CCI-injured mice compared to the contralateral side. Differences were evaluated with Mann-Whittney *U* test among experimental groups for each time-point. Experimental groups shown in the graphs include both female and male mice, with data from both sexes combined for the analysis. ∗p ​< ​0.05, ∗∗p ​< ​0.01, ∗∗∗p ​< ​0.001 and ∗∗∗∗p ​< ​0.0001, respectively. CCI- controlled cortical impact.

Three sections per animal were used, corresponding to bregma levels −0.9 ​mm, −1.4 ​mm and −1.9 ​mm. The observer conducting the analysis was blinded to the treatment status of the sections that were being assessed. Sections were visualized using an Olympus BX51 light microscope in 4x magnification. A stitched image was acquired for each section by using the Multiple Image Alignment (MIA) manual function in the CellSens digital imaging software. Images were imported into ImageJ and a free-hand selection of the ipsilateral and contralateral cortical hemisphere and corpus callosum/external capsule were outlined, respectively, as shown in Fig. 2B. The relative tissue fraction loss for either gray matter or white matter was defined as the ratio of the difference between the area of the contralateral and the ipsilateral selection to the area of the contralateral selection ([A_C_ - A_I_]/A_C_). Tissue loss percentages were averaged between the three sections.

### Immunofluorescence, image acquisition and quantification analysis

Sections were chosen between bregma levels −1.1 ​mm to −1.7 ​mm and were initially washed three times in PBS for 10 ​min per wash. All sections underwent heat-induced antigen retrieval in citrate buffer (10 ​mM, pH 6.0) at 95 ​°C for 35 ​min. Blocking of sections was done in PBS-T (PBS with 0.05 ​% Triton-X100) containing 3 ​% Normal Donkey Serum (NDS). Incubation with primary antibodies was performed in blocking solution at 4 ​°C overnight. Primary antibodies used included a mouse monoclonal anti-Neurofilament H, SMI-31 (1:500, BioLegend, USA), a mouse monoclonal anti-APC (CC1) (1:200, Calbiochem, USA), a goat polyclonal anti-Olig2 (1:300, R&D Systems, USA), a mouse monoclonal anti-PCNA (1:1000, Merck Millipore, USA) and a rabbit polyclonal anti-PDGFRα (1:300, Cell Signaling Technology, USA). Following incubation with the primary antibody, sections were washed three times in PBS and thereafter, incubated in PBST containing the fluorophore-conjugated secondary antibodies for 1 ​h. After washing with PBS, counter-staining of sections with DAPI (1:2000) was done in PBS for 15 ​min, followed by two final washes with PBS and mounting of sections on slides using PVA-DABCO (Sigma-Aldrich, USA).

**Fig. 3:**
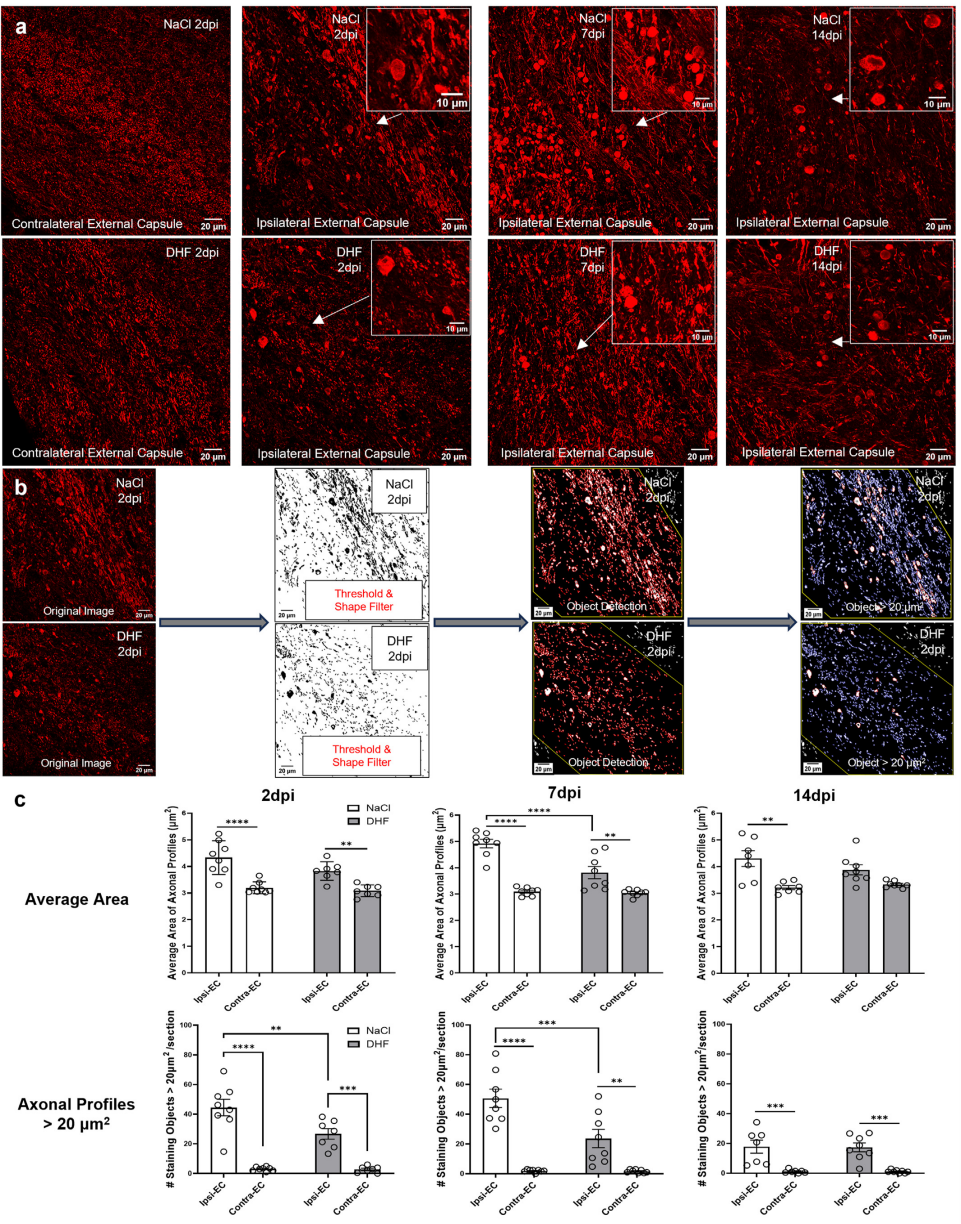
**7,8-DHF prevents axonal swelling at 2 and 7 dpi as visualized by SMI-31 immunofluorescent staining**. **(A)** Representative confocal 40x magnification, maximally projected z-stack SMI-31 images from the ipsilateral and contralateral external capsule of CCI-NaCl and CCI-DHF mice at 2, 7 dpi and 14 dpi. Insets depict a higher magnification image of the area where axonal swellings are located. **(B)** Representative images from the semi-automatic quantification workflow depicting the axonal staining object detection applied on binary images which underwent intensity thresholding and shape filtering (ImageJ). Object detection and assessment of object size was done in QuPath. **(C)** Quantification of the average area of SMI-31 immunofluorescent axonal profiles and of axonal profiles with an area larger than 20 ​μm^2^ in the ipsilateral and contralateral external capsule at 2, 7 dpi and 14 dpi, respectively. Experimental groups shown in the graphs include both female and male mice, with data from both sexes combined for the analysis. ∗p ​< ​0.05, ∗∗p ​< ​0.01, ∗∗∗p ​< ​0.001 and ∗∗∗∗p ​< ​0.0001, respectively. CCI- controlled cortical impact.

For the quantification of immunofluorescent axonal swellings, confocal imaging was done using a Leica SP8 system (Leica Microsystems, Wetzlar, Germany). Two images of the peri-lesional external capsule, one image of the corpus callosum and one image of the contralateral external capsule were taken in 40x magnification. 18 ​μm z-stack images were acquired with a step size of 1.1 ​μm on a 1024x1024 resolution for subsequent analysis. Semi-quantitative analysis was done using ImageJ (Billerica, MA, USA) and QuPath 0.5.1 [60]. Maximally projected images were exported from the Leica Software and imported to ImageJ for single-channel thresholding processing (“Otsu” method). Afterwards, binary images were processed with the “Shape Filter” plugin (Area: 0.005 – Infinity) in order to apply a cut-off value for very small size staining objects. Images were then imported to QuPath for object detection and analysis. A free-hand selection was used to define the region of interest (ROI) which included the depicted external capsule and a portion of the gray-white matter junction in the lower cortical layer. Identification of objects in the desired ROI was achieved by using the “Cell Detection” command with the same parameters for each image (Requested pixel size: 0.1 ​μm, Background Radius: 8 ​μm, Median Filter Radius: 0, Sigma: 0.5 ​μm, Min-Max Area: 0–400 ​μm^2^, Threshold Intensity: 1). Morphometric measurements of the detected objects were collected, and a size threshold was applied for selection of objects with an area size larger than 20 ​μm^2^. An overview of the image processing workflow is depicted in Fig. 3B. Data acquired from the same region were averaged between the two chosen sections.

For quantification of PCNA/PDGFRα/Olig2-positive cells, triple immunofluorescence was visualized with confocal microscopy. Two 20x images, 13 ​μm z-stack sized, were acquired from the ipsilateral external capsule, one image from the corpus callosum and one image from the external capsule. Additionally, one image was taken from the corpus callosum of sham-operated mice and two images of the external capsule bilaterally. Data acquired from the same region were averaged between two chosen sections.

### Terminal deoxynucleotidyl transferase dUTP nick end labelling (TUNEL)

Labeling of cells with terminal deoxynucleotidyl transferase dUTP was done with a Click-iT™ TUNEL Colorimetric IHC Detection Kit (Invitrogen, Thermo Scientific, MA, USA). Free-floating sections were not pre-treated with Proteinase K but underwent heat-induced antigen retrieval in citrate buffer instead in order to be used for immunofluorescence labelling further on. The terminal deoxynucleotidyl transferase reaction was done with 1/3 of the enzyme concentration recommended by the manufacturer. Sections were incubated for 30 ​min at 37 ​°C in the TUNEL reaction mixture. Visualization of incorporated dUTP was achieved via a copper-catalyzed click reaction with the use of a streptavidin-conjugated Alexa 488 fluorophore. Incubation with the streptavidin-conjugated fluorophore was done is PBST for 1 ​h at 4 ​°C.

Two sections per animal, ranging from distance to bregma −1.1 ​mm to −1.7 ​mm, were chosen and analyzed using confocal microscopy. Two 20x z-stack images were taken from the ipsilateral external capsule, one image from the corpus callosum and one image from the contralateral external capsule. Quantification of TUNEL-positive CC1-positive cells was done on 20x images z-stack images, 16 ​μm stack size, which were taken at a resolution of 1024x1024. For assessment of apoptotic nuclei and representative images, 40x images were taken with a z-stack of 18 ​μm. Data acquired from the same region were averaged between chosen sections.

### Statistics

Statistical analysis and graphs were made with GraphPad Prism 9 (GraphPad Software, La Jolla, CA, USA). Tissue loss percentage data were a priori, due to the relatively low animal number per treatment group, treated as non-parametric data and tested with Mann–Whitney *U* test for differences between treated and untreated experimental groups. Non-parametric data are presented as median with the interquartile range. Axonal profiles area data and PCNA/PDGFRα/Οlig2+ cell count data were tested for normality with the Shapiro-Wilk test and visual assessment of the Normal QQ plot of the residuals. Both sets of data were normally distributed, and statistical comparisons were made using a Two-Way ANOVA for each time point, followed by Tukey-multiple comparison tests. Data are depicted as mean ​± ​standard error of the mean (SEM). TUNEL-positive cell counts were not normally distributed, thus a Kruskal-Wallis test was used to determine statistical differences in the distributions of the experimental groups, followed by Dunn's multiple comparisons test. TUNEL data are, thus, presented as median with the interquartile range.

## Results

### 7,8-DHF preserves white matter tissue without affecting cortical tissue loss following CCI

To evaluate brain tissue loss with or without treatment with 7,8-DHF following CCI, we histologically assessed coronal sections using Luxol Fast Blue (LFB)/Nissl staining (Fig. 2a). The purpose of this double histological stain was to specifically dissect the gray and white matter compartment of the lesion and quantify the percentage loss of the ipsilateral cortex and external capsule, respectively, to their contralateral counterparts (Fig. 2b). No quantification was conducted on brain sections of sham-injured mice since no lesion was evident. Quantification of Nissl-stained gray matter revealed no effect of 7,8-DHF on hemispheric cortical tissue loss at 2, 7 and 14 days post-injury (dpi) between CCI-DHF and CCI-NaCl mice (Fig. 2c). Conversely, quantification of the LFB-stained area revealed a preservation of white matter tissue of CCI-DHF mice at 2 and 7 dpi (Fig. 2c). No difference could be observed at 14 dpi. Below the lesion site, the external capsule was morphologically altered by the injury (Fig. 2a). Comparison of tissue loss percentage between male and female animals is shown in Table 1. In view of the absence of statistical differences, the results of female and male mice were grouped together for the analyses. Overall, these results point towards an attenuated injury to the peri-lesional white matter of DHF-treated, brain-injured animals without an effect on neuronal loss in the lesioned cortex.

**Table 1:** **Tissue loss following TBI in male and female mice following saline or 7,8-DHF treatment**. Tissue loss percentage is presented as median with the interquartile range (IQR). Statistical comparisons were made between the 2 groups of the same treatment and tested with the Mann–Whitney *U* test. No significant sex differences were observed.

Tissue Loss % ​± ​(SD)	2 dpi		7 dpi		14 dpi	
	GM	WM	GM	WM	GM	WM
Female- NaCl	13.6 (5.5–21.8) (n ​= ​4)	12.6 (5.2–15) (n ​= ​4)	12.6 (8.1–16.2) (n ​= ​4)	16.5 (6.2–22.3) (n ​= ​4)	16.8 (9.3–19.1) (n ​= ​4)	7.1 (3.1–10.9) (n ​= ​4)
Male-NaCl	16.3 (7.9–25.3) (n ​= ​4)	17.3 (10.3–24.9) (n ​= ​4)	21 (14.8–25.6) (n ​= ​4)	11.1 (5.2–15.9) (n ​= ​4)	17.2 (2.7–31.9) (n ​= ​3)	4.5 (1.6–36.2) (n ​= ​3)
Female-DHF	5.5 (2.2–12.7) (n ​= ​4)	3.6 (0.6–4.3) (n ​= ​4)	4.8 (2.2–8.5) (n ​= ​4)	3.7 (1.6–7.2) (n ​= ​4)	13.7 (10–16.1) (n ​= ​4)	0.7 (−1.4–2.6) (n ​= ​4)
Male-DHF	22.8 (13–23.1) (n ​= ​3)	1.7 (1.7–6) (n ​= ​3)	20 (9.2–27.6) (n ​= ​4)	3.9 (0.01–16.7) (n ​= ​4)	12.3 (7–15.9) (n ​= ​4)	6.6 (3–16.9) (n ​= ​4)

Images obtained from the coronal sections, histologically evaluated by LFB/Nissl Staining and used for the quantification of tissue loss, can be accessed in the Open-Source Repository Figshare at https://doi.org/10.6084/m9.figshare.26984440.v1. Additionally, the body weight dynamics for both treated and untreated CCI-mice are shown in Supplementary Fig. 1. Following CCI, brain-injured mice had a transient weight loss that was similar in both injury groups. Aged male mice had higher body weight compared to their female counterparts, both prior and post-injury. No changes in body weight were observed in either treated or untreated sham-injured mice.

### 7,8-DHF reduces SMI-31 positive axonal swellings within the ipsilateral external capsule at 2 and 7 dpi

The attenuated white matter injury by 7,8-DHF treatment, in combination with the morphological alterations seen in the ipsilateral external capsule, prompted us to visualize axonal profiles in the subcortical white matter with the use of an immunofluorescent staining protocol for SMI-31. This antibody targets a phosphorylated epitope in the Neurofilament heavy chain protein (pNfH). SMI-31 immunostaining revealed extensive axonal swellings within the ipsilateral external capsule at both 2 dpi and 7 dpi and, to a lesser extent, at 14 dpi (Fig. 3a). Axonal profiles consisted of a mixture of retraction bulbs, varicose axonal profiles and beading morphologies (Fig. 3a). Axonal swellings were mostly observed below the lesion site, indicating a regional-specific pathology which may be caused by the focal sheer stress applied in this experimental TBI model. Few swellings were observed in the external capsule medial to the lesion site, while no swellings could be observed in the corpus callosum (data not shown). Additionally, no swellings were observed in either the contralateral external capsule (Fig. 3a) or the white matter of sham-injured mice (data not shown).

In order to evaluate changes in the SMI-31 staining pattern by the treatment, we performed a semi-automatic quantitative analysis on the axonal swellings of the ipsilateral external capsule, as visualized by immunofluorescence. We focused our analysis on 2 and 7 dpi due to the beneficial effect of 7,8-DHF seen on the extent of white matter pathology at these two time-points. Axonal staining profiles in the ipsilateral capsule showed an increased mean axonal area when compared to those located in the contralateral external capsule (Fig. 3c). This difference was observed in both CCI-DHF and CCI-NaCl mice. These relative differences were consistent at both 2 and 7 dpi. There was a significant attenuation of this TBI-related increase in the white matter of 7,8-DHF-treated mice at 7 dpi but not 2 dpi (Fig. 3c). This result indicates that 7,8-DHF may promote resolution of axonal swellings in a time-dependent manner following TBI.

To further analyze the mechanisms of 7,8-DHF treatment, we applied a size threshold in order to specifically quantify the axonal swellings. Following a preliminary assessment of the staining pattern among different sections of brain-injured mice, we chose 20 ​μm^2^ as the cut-off point and quantified the staining profiles within the regions of interest. Axonal staining profiles larger than 20 ​μm^2^ were present in the ipsilateral capsule of both treated and untreated CCI-mice and absent in the contralateral external capsule (Fig. 3a). CCI-DHF mice exhibited a significantly lower number of such staining profiles in the subcortical white matter and a partially preserved SMI-31 staining pattern at both 2 and 7 dpi compared to CCI-NaCl mice (Fig. 3d). No sex-dependent differences were observed in any of the brain-injured groups and data were grouped for morphometric analysis of the axonal profiles. Collectively, these results suggest that 7,8-DHF may exert a protective effect on the peri-contusional white matter through mitigation of axonal damage and alleviation of axonal swelling.

In accordance with the white matter tissue loss assessment at 14 dpi using LFB, we did not observe a late effect of DHF treatment on the axonal profiles of the ipsilateral external capsule at 14 dpi (Fig. 3c). Axonal swellings were still present in the perilesional white matter at 14 days following TBI, albeit with a lower overall number of axonal profiles larger than 20 ​μm^2^ when compared to 2 and 7 dpi (Fig. 3c). These data provide further evidence that axonal pathology may be causally linked to white matter tissue loss and, ultimately, progressive white matter atrophy in the post-injured brain.

### 7,8-DHF reduced post-injury death to oligodendroglia

**Fig. 4:**
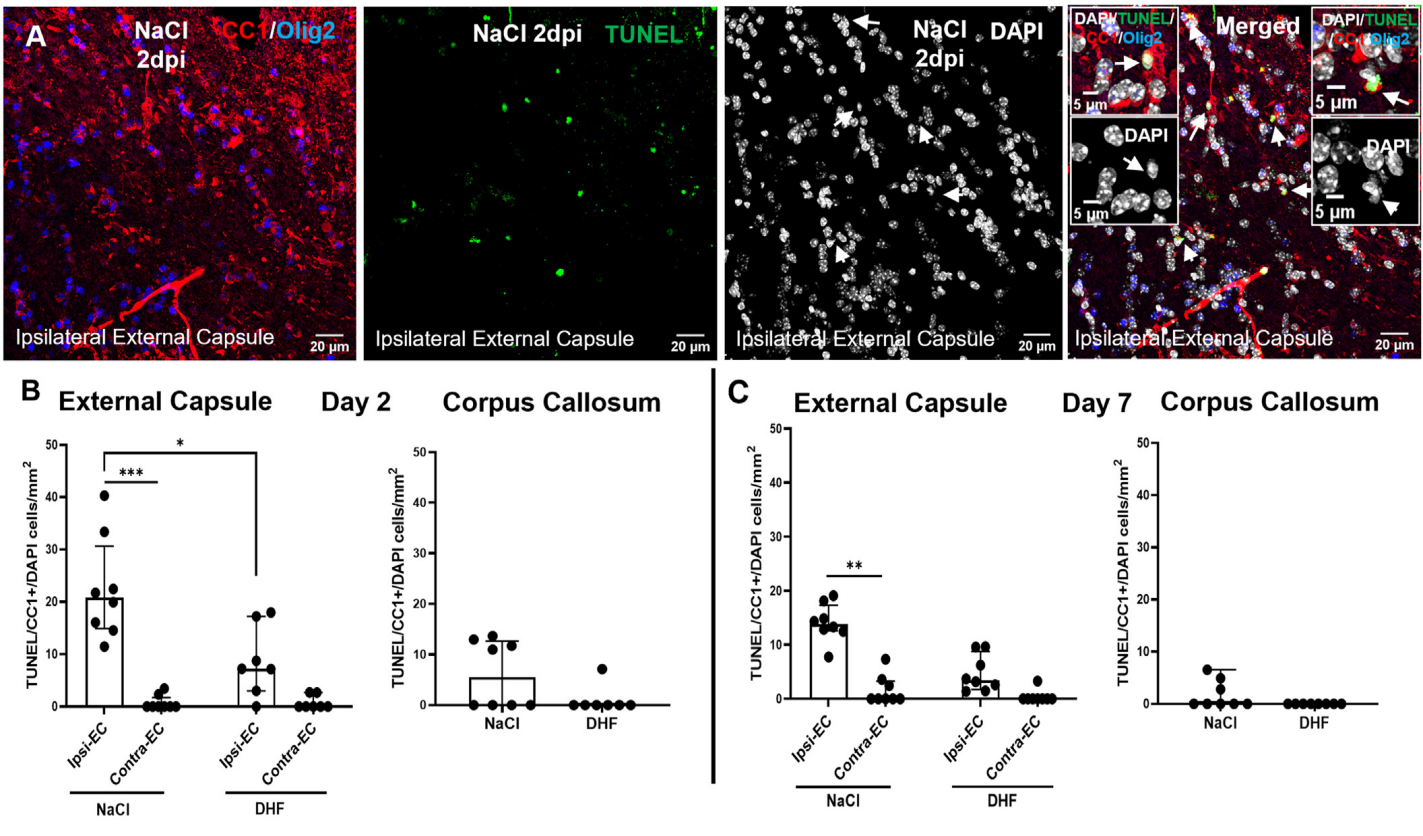
**7,8-DHF attenuates oligodendrocyte loss following TBI in aged mice**. **(A)** Representative confocal 40x images of combined immunofluorescent staining of Olig2/CC1/TUNEL/DAPI channels depicting TUNEL/CC1-positive cells within the ipsilateral external capsule of a CCI-injured mouse. TUNEL/CC1+ cells show no nuclear Olig2 immunoreactivity in the Olig2 channel. Arrows in the DAPI channel and in the pseudo-colored merged image point to apoptotic cells with irregular nuclear morphologies located below the lesion site. Insets depict higher magnification images of apoptotic oligodendroglial nuclei including all pseudo-colored channels as well as DAPI channel alone. **(B, C)** Quantification of TUNEL/CC1+ cells in the ipsilateral and contralateral external capsule as well as the corpus callosum of CCI-NaCl and CCI-DHF mice at 2 and 7 dpi, respectively. Statistical differences were determined by Kruskal-Wallis test followed by Dunn's multiple comparisons test. Experimental groups shown in the graphs include both female and male mice, with data from both sexes combined for the analysis. ∗p ​< ​0.05, ∗∗p ​< ​0.01, ∗∗∗p ​< ​0.001 and ∗∗∗∗p ​< ​0.0001, respectively. CCI- controlled cortical impact.

Our next step was to visualize apoptotic oligodendroglia in the peri-lesional white matter and determine whether treatment with 7,8-DHF attenuated oligodendrocyte loss following CCI. By applying TUNEL, we could detect a small number of TUNEL-positive cells in the ipsilateral external capsule at 2 dpi in both CCI-DHF and CCI-NaCl mice (Fig. 4). TUNEL-positive cells were abundant in the peri-lesional cortex and were used as a positive control for the TUNEL reaction (data not shown). Most of the TUNEL-positive cells detected in the white matter exhibited partial cytosolic immunoreactivity for CC1, indicating that such cells are apoptotic oligodendrocytes (Fig. 4a). Comparison with non-TUNEL positive cells within the same field of view revealed that apoptotic TUNEL-positive CC1+ cells did not exhibit nuclear immunoreactivity for Olig2 (Fig. 4a). Nuclear morphological profiles of such cells included pyknotic nuclei with a smaller size when compared to seemingly healthy nuclei in the vicinity, fragmented nuclei as well as DAPI profiles with dense and diffused staining of chromatin.

We proceeded to quantify apoptotic oligodendroglia in the peri-contusional white matter. Due to potential non-specific labelling of the TUNEL protocol, we only quantified cells with marked TUNEL-associated nuclear immunofluorescence, distinct nuclear morphologies, as previously described, and cytosolic CC1+ immunoreactivity. Quantification of TUNEL/CC1+ cells revealed a small TBI-related increase of apoptotic oligodendrocytes in the ipsilateral white matter of brain-injured, saline-treated mice when compared to the same contralateral regions at 2 dpi (Fig. 4b). Comparison of TUNEL/CC1+ cells in the ipsilateral external capsule of CCI-NaCl and CCI-DHF mice revealed a significant difference between the two experimental groups at 2 dpi, indicating an attenuation of oligodendrocyte death by 7,8-DHF treatment in the perilesional white matter (Fig. 4b). TUNEL/CC1+ cells were rare in the contralateral external capsule as well as the corpus callosum (Fig. 4a and b). Quantification of TUNEL/CC1+ cells in the ipsilateral external capsule at 7 dpi revealed a persistent significantly higher apoptotic cell number compared to the contralateral region of CCI-NaCl mice (Fig. 4c). Overall, these results argue for a protective effect of 7,8-DHF on oligodendrocyte death. However, oligodendrocyte loss did not seem to be the main cause of white matter pathology in the sub-acute phase of the CCI model.

### 7,8-DHF does not affect reactive proliferation of PDGFRα+/Olig2+ cells post-injury

**Fig. 5:**
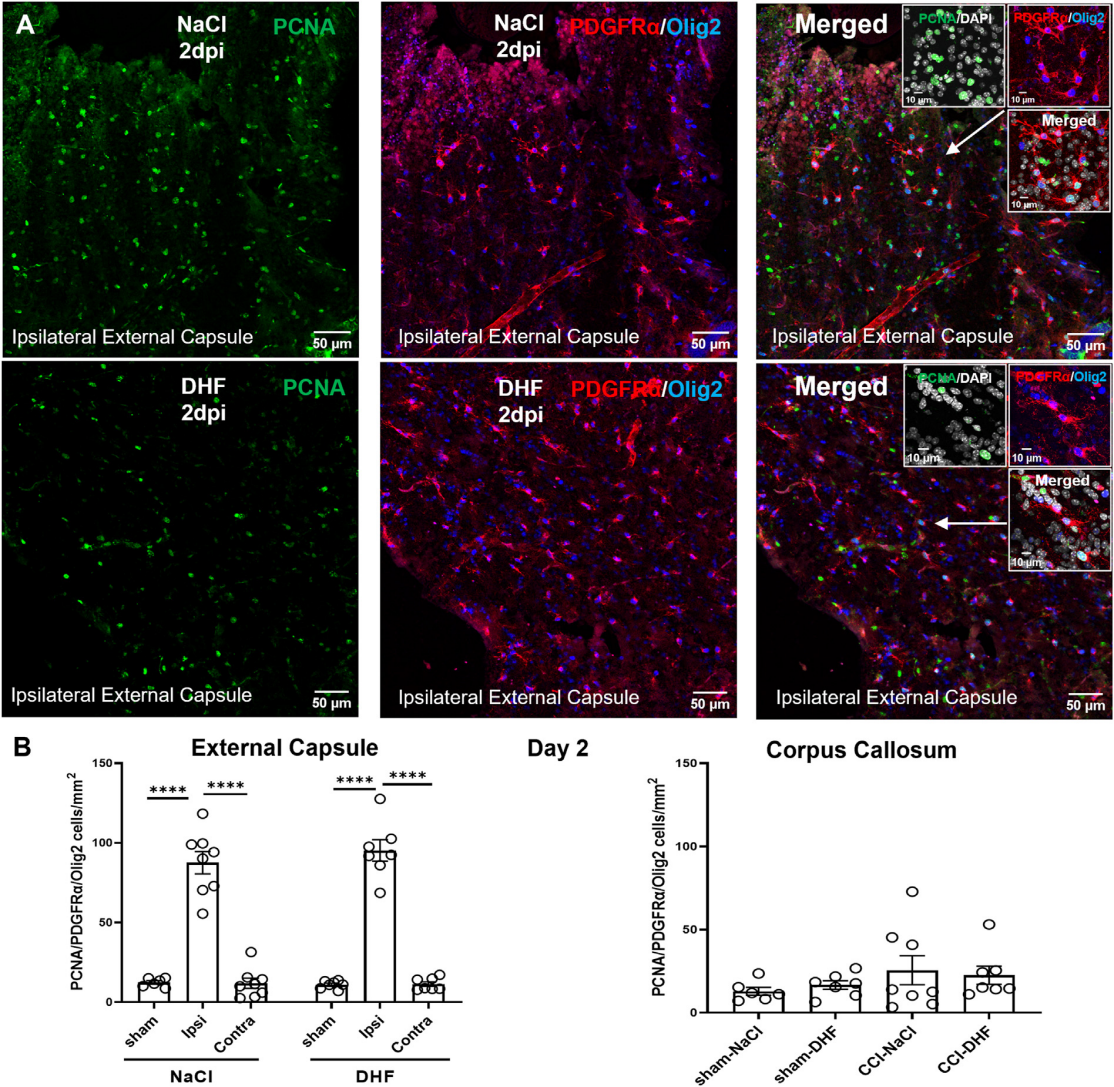
**Post-injury treatment with 7,8-DHF does not alter post-injury proliferation of oligodendrocyte precursor cells in the white matter**. **(A)** Representative confocal 20x images of triple immunofluorescent staining for PCNA/PDGFRα/Olig2 in the ipsilateral external capsule of CCI-NaCl and CCI-DHF mice. Insets depict confocal 40x images from the region the arrows point to. **(B)** Quantification of PCNA/PDGFRα/Olig2+ in the ipsilateral and contralateral external capsule as well as the corpus callosum of CCI-NaCl and CCI-DHF mice at 2 dpi. Statistical differences were determined by Two-Way ANOVA followed by Tukey's multiple comparisons test. Experimental groups shown in the graphs include both female and male mice, with data from both sexes combined for the analysis. ∗p ​< ​0.05, ∗∗p ​< ​0.01, ∗∗∗p ​< ​0.001 and ∗∗∗∗p ​< ​0.0001, respectively. CCI- controlled cortical impact.

The plasticity-enhancing effects of 7,8-DHF in the post-injured brain may involve the recruitment of oligodendrocyte precursor cells (OPCs) to the lesion site. We, thus, hypothesized that 7,8-DHF may affect reactive proliferation of PDGFRα+ OPCs in the injured white matter. We combined triple immunofluorescent staining for PCNA, a proliferation marker expressed during DNA replication, with Olig2 and platelet-derived growth factor receptor a (PDGFRα), protein markers associated with oligodendroglial progenitors. We quantified triple PCNA/PDGRFα/Olig2-positive cells in the peri-lesional white matter, the corpus callosum, the contralateral external capsule as well as in the white matter of sham-NaCl and sham-DHF mice. Brain-injured mice exhibited a significant TBI-related increase of PCNA/PDGRFα/Olig2+ cells in the ipsilateral external capsule compared to both the contralateral external capsule and the external capsule of sham-injured mice (Fig. 5). Sham-injured mice showed very low numbers of PCNA/PDGRFα/Olig2+ cells in their white matter, with levels similar to those observed in the contralateral external capsule of CCI-mice (Fig. 5b). No difference could be observed between the external capsule of CCI-NaCl and CCI-DHF mice as well as between sham-NaCl and sham-DHF mice (Fig. 5b). Furthermore, no difference in cell numbers could be observed among the experimental groups in the corpus callosum (Fig. 5b). These results indicate that 7,8-DHF does not alter post-injury proliferation of PDGFRα+ OPCs in the white matter of aged mice.

## Discussion

Despite the rapidly increasing number of elderly patients with TBI, treatment options to date are limited to surgical evacuation of mass lesions, supportive treatments targeting TBI-induced symptoms, and rehabilitation. Overall, prognosis after TBI is poor in the elderly and novel treatments have rarely been evaluated in the aging rodent brain. As such, there is a critical need for more research focused on geriatric TBI, as the pathophysiological responses and potential therapeutic targets can vary remarkably with age. In the present study, we evaluated the treatment effect of 7,8-DHF, a small BDNF-mimetic molecule, on white matter pathology following focal TBI in 23-month-old, a very advanced, age in mice. Our data provide evidence that 7,8-DHF, a compound extensively studied in the context of neurodegenerative diseases, can exert a protective effect on the injured white matter of the aging rodent brain through mitigation of axonal damage and loss of mature oligodendrocytes.

The neuroprotective efficacy and plasticity-enhancing effects of 7,8-DHF have been previously documented in models of acute brain injury, including a reduction in infarct volumes in a transient middle cerebral artery occlusion model of ischemic stroke, at a 7,8-DHF dose of 5 ​mg/kg prior to stroke induction [46]. In experimental TBI models, post-injury administration of 7,8-DHF at a dose of 5 ​mg/kg did not affect lesion size in a mouse model of CCI injury [50], while higher dosages such as 20 ​mg/kg did reduce contusion volume [48], indicating a dose-dependent efficacy of 7,8-DHF. In our present study, cortical tissue loss was not reduced by 5 ​mg/kg of 7,8-DHF treatment, confirming previous reports in young brain-injured mice [50]. However, we observed a specific effect of 7,8-DHF on the injured white matter at 2 and 7 dpi, but not 14 dpi. It should be noted that dosing, the number of injections as well as the evaluated time window can influence the neuroprotective efficacy of 7,8-DHF, which needs to be further explored in additional studies. Continuous treatment with mini pumps over a longer duration, or repeated injections for extended survival time-points, may further uncover the beneficial effects of 7,8-DHF.

White matter disruption is commonly observed in TBI and progressive white matter atrophy accounts for persistent cognitive impairment in the chronic phase of TBI [33,61,62]. Both diffuse and focal damage can lead to disconnection of white matter regions in the brain's network, leading to behavioral alterations [63]. Structural, cellular and functional changes have been reported in the aging white matter [35,38,39,64]. Such changes may indicate that the aged white matter is more prone to degeneration following brain injury. Furthermore, the beneficial functions of neurotrophins, including BDNF, have been shown to decline in the aging brain [54,56,57,65]. TBI results in axonal pathology, and it is speculated that age may further exacerbate axonal degeneration leading to worse outcomes [66].

Several studies provide evidence that 7,8-DHF may influence plasticity mechanisms that are associated with functional recovery. In specific, improved neurobehavioral outcome was observed in experimental studies involving cognitively impaired aged rats, 5XFAD Alzheimer mouse models, rodent models of TBI, cuprizone-induced demyelination, epilepsy-inducing models, Parkinson models as well chronic exposure to alcohol [46,[48], [49], [50], [51],[67], [68], [69], [70], [71], [72], [73], [74]]. Several plausible mechanisms have been suggested for the action of 7,8-DHF including regulation of pro-/anti-apoptotic signals leading to attenuated cell death as well as increased neuronal plasticity mediated by GAP43, synapsins and activation of CREB signaling [46,[48], [49], [50], [51],[67], [68], [69], [70], [71], [72], [73], [74]]. A protective effect was specifically found on the dendritic spines of the peri-lesional cortical neurons without affecting cortical lesion size following the CCI model [50]. This finding is consistent with the results of our present study. Following the CCI model, we observed a reduction of SMI-31-positive axonal swellings due to treatment with 7,8-DHF. Axonal swellings most probably reflect an accumulation of phosphorylated Neurofilament Heavy chain molecules in the sites of axonal damage. Treatment with 7,8-DHF may, in turn, alleviate axonal swelling by regulating proteins associated with axonal retrograde and anterograde transport. Taken together, 7,8-DHF, and ultimately TrkB signaling, may promote plasticity through attenuation of axonal swelling and preservation of axonal integrity, processes which can be beneficial for behavioral recovery following brain injury.

We also investigated the effects of 7,8-DHF on a second white matter component following TBI, the oligodendroglial cells. Oligodendroglial cells express the TrkB receptor [[75], [76], [77]]. These receptors have been shown to be upregulated, even 8 weeks, following experimental TBI, suggesting the possibility of their modulation in the chronic phase of TBI [[78], [79], [80]]. Human TBI has been reported to result in oligodendrocyte death [26]. Several studies have demonstrated immunoreactivity for cleaved caspase 3 (CC3) in oligodendrocytes following experimental models of TBI [[27], [28], [29]]. Conversely, in a closed head diffuse midline TBI mouse model, there was no increase of TUNEL ​+ ​oligodendroglia in the corpus callosum [25]. TUNEL and CC3 immunoreactivity may depict different oligodendroglial apoptotic stages with the first indicating late-stage and the second earlier stages of apoptosis. Regulation of oligodendrocyte apoptosis may also vary among experimental rodent models of TBI, with different levels of injury severity, and may exhibit species-related differences.

In our study, we observed a significant reduction by 7,8-DHF on the number of TUNEL-positive oligodendrocytes in the perilesional white matter. However, the number of TUNEL-positive oligodendrocytes was relatively low, and acute oligodendrocyte apoptosis may have occurred prior to 2 dpi. Taken together, attenuated oligodendrocyte death, in combination with the reduction in axonal swellings observed by 7,8-DHF treatment, could lead to reduced atrophy of white matter tracts [24,33,81,82]. Maintenance of axonal integrity in conjunction with preservation of oligodendrocyte viability, as suggested by 7,8-DHF treatment in the acute/sub-acute phase of TBI, may thus prevent long-term white matter loss after TBI [83].

We did not detect sex-related differences in response to TBI and treatment with regards to tissue loss and axonal pathology post-TBI. It is established that aged male mice have higher body weight compared both to younger male mice and female mice [84], a weight pattern also seen in our study. Increased body weight is linked to worse outcomes and aggravated pathology [85,86]. Additionally, emerging sex differences in both the human and experimental setting where worse clinical outcomes in females, yet improved outcomes in female animals, have been suggested [87]. The incidence of TBI among elderly women is increasing rapidly [7], thus, necessitating further investigation on sex-related differences in TBI.

Post-injury proliferation of OPCs has been previously studied [36,[88], [89], [90], [91], [92], [93]], and following ischemic stroke in mice BDNF administration resulted in increased OPC proliferation [94]. In addition, administration of 7,8-DHF and TrkB agonist LM22A-4 promoted the repopulation of oligodendrocytes in the corpus callosum in a cuprizone demyelination model [74,95], while in vitro administration of BDNF in OPC cultures promoted OPC proliferation [77]. These findings indicate a role for BDNF in oligodendrogenesis.

We previously reported a reduced number of proliferating OPCs in the aging brain following TBI [36]. In the present study, the OPC number was not influenced by 7,8-DHF treatment. Senescent OPCs may exhibit diminished proliferative capabilities [[96], [97], [98], [99]]. Additionally, the effect of 7,8-DHF, and ultimately TrkB signaling, may be associated with the differentiation of OPCs rather than with their proliferation. We previously reported an accumulation of proliferating PDGFRα+ OPCs in the aging white matter [36]. This finding, in combination with other studies, confirms that aged OPCs differentiate slowly and might be unresponsive to pro-differentiation molecular ques [[96], [97], [98],^100^]. Our results argue that the effects of 7,8-DHF following TBI are primarily mediated through reduction of axonal and mature oligodendrocyte pathology, not at the OPC stage.

Our study is not without limitations. We did not include behavioral assessment of the included mice due to their very high age and weight, posing difficulties in conducting e.g. cognitive and neurological motor tests. Additionally, the dosing and therapeutic time window require further optimization. We chose the 5 ​mg/kg dose in our study, since behavioral improvement was previously observed with this dose and since it may be more clinically relevant than the 20 ​mg/kg dose. Finally, since the CCI model could be characterized as a severe focal injury model, additional studies using models with less overt tissue damage would be of benefit to unveil the potential therapeutic capabilities of 7,8-DHF.

Preclinical studies on TBI in old mice in combination with an adjuvant treatment are limited. This study evaluated 23-month-old mice and provided evidence of a therapeutic impact of 7,8-DHF, a BDNF-mimetic compound and TrkB agonist, on white matter pathology in an experimental model of focal TBI. Administration of 7,8-DHF resulted in attenuated axonal damage and oligodendrocyte loss but did not affect reactive OPC proliferation and cortical tissue loss. It would be of relevance for future studies to focus on how 7,8-DHF, and ultimately TrkB agonism, mechanistically influences axonal pathology and white matter preservation following TBI and how such processes can be associated with functional recovery, especially in the case of the aging brain.

## Author Contributions

**Georgios Michalettos:** Visualization, Methodology, Project Administration, Software, Data Curation, Formal Analysis, Writing – Original Draft, Writing – Review and Editing. **Fredrik Clausen:** Methodology, Project Administration, Software, Data Curation, Review and Editing. **Elham Rostami:** Methodology, Investigation, Conceptualization, Project Administration, Funding Acquisition, Resources, Supervision, Validation, Writing - Review and Editing. **Niklas Marklund:** Methodology, Investigation, Conceptualization, Project Administration, Funding Acquisition, Resources, Data Curation, Formal Analysis, Supervision, Validation, Writing – Original Draft, Writing – Review and Editing.

## Data Availability Statement

All data presented and analyzed in this study are included in the present article. All requests for raw data may be sent to the corresponding author.

## Funding

This work was funded by the Swedish Brain Foundation, the Hans-Gabriel af Trolle-Wachtmeister Foundation (HGATW), the Alborada Trust and Hospital ALF funds, Marianne and Marcus Wallenberg foundation, The ALF agreement, and Kjell and Märta Beijer Foundation.

## Declaration of Competing Interest

The authors declare no conflict of interest.
