# Supplementary material for: Post-injury treatment with 7,8-dihydroxyflavone attenuates white matter pathology in aged mice following focal traumatic brain injury
Source: Neurotherapeutics. 2024 Oct 20;22(1):e00472. doi: 10.1016/j.neurot.2024.e00472 (PMC11742853; doi:10.1016/j.neurot.2024.e00472)
Supplement: Multimedia component 1 [file mmc1.docx]

**Post-injury treatment with 7,8-Dihydroxyflavone attenuates white matter pathology in aged mice following focal traumatic brain injury**

Georgios Michalettos^1^, Fredrik Clausen^2^, Elham Rostami^2,3^, Niklas Marklund^1,4^

^1^ Lund Brain Injury Laboratory for Neurosurgical Research, Department of Clinical Sciences, Neurosurgery, Lund University, Lund, Sweden

^2^ Department of Medical Sciences, Section of Neurosurgery, Uppsala University, Uppsala

^3^ Department of Neuroscience, Karolinska institute, Stockholm

^4^ Department of Clinical Sciences Lund, Neurosurgery, Lund University and Lund University Hospital, Lund, Sweden

Supplementary Material

**Corresponding author:** Niklas Marklund

Skåne University Hospital EA-blocket plan 4

Entrégatan 7, 222 42 Lund, Sweden

[niklas.marklund@med.lu.se](mailto:niklas.marklund@med.lu.se)

+46 725 95 02 61

0000-0002-9797-5626


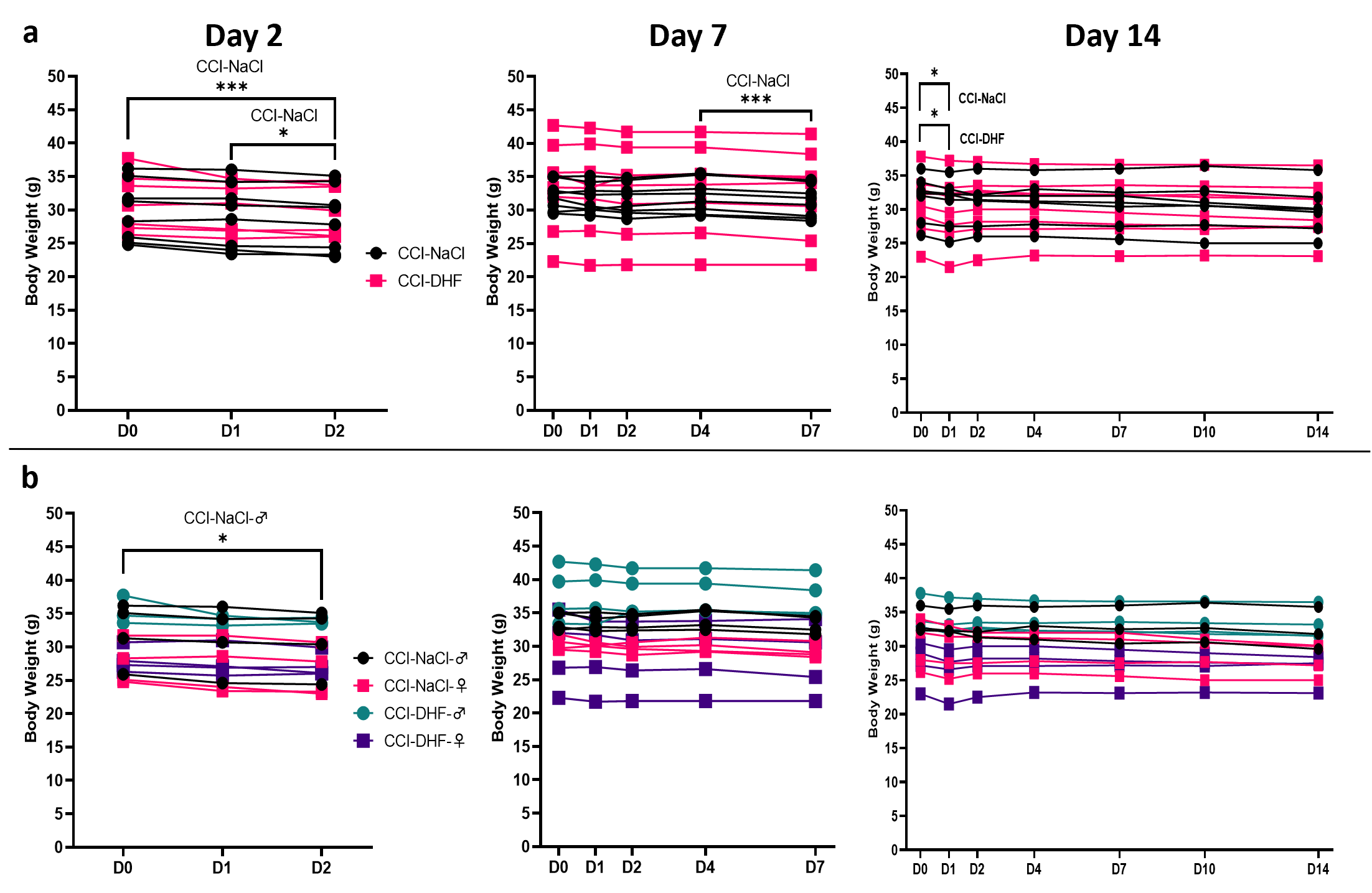


**Supplementary Figure 1 Body weight of DHF-treated and saline-treated 23-month-old mice following CCI (A)** Body weight dynamics of CCI-NaCl and CCI-DHF mice measured separately on 2, 7 and 14 dpi experimental groups. CCI mice tend to lose weight the first two days following injury and recover their weight thereafter. As seen in 2dpi and 7 dpi measurements, CCI-NaCl exhibited, on average, a significant drop in their body weight. A significant decrease in weight was observed for both 14dpi CCI-NaCl and CCI-DHF mice, one day following CCI. Experimental groups shown in the graphs include both female and male mice, with data from both sexes combined for the analysis. **(B)** Body weight dynamics of CCI-NaCl and CCI-DHF mice measured separately on 2, 7 and 14 dpi experimental groups, segregated by sex, as indicated by different coloring. A significant drop in body weight was observed for 2dpi male CCI-NaCl mice at day 2 compared to day 0. Statistical differences were determined by repeated measures Two-Way ANOVA followed by Sidak’s multiple comparisons tests. * p < 0.05, ** p < 0.01, *** p < 0.001 and **** p < 0.0001, respectively. CCI- controlled cortical impact
